# Apelin receptor inhibition in ischemia-reperfused mouse hearts protected by endogenous n-3 polyunsaturated fatty acids

**DOI:** 10.3389/fphar.2023.1145413

**Published:** 2023-10-24

**Authors:** Shuang Zheng, Weijiang Tan, Xiang Li, Lijing Wang, Caiyi Zhu, W. Glen Pyle, Jianxin Chen, Jian Wu, Xuecong Ren, Honghua Chen, Yunzeng Zou, Peter H. Backx, Feng Hua Yang

**Affiliations:** ^1^ Guangdong Laboratory Animals Monitoring Institute, Guangzhou, China; ^2^ College of Veterinary Medicine, South China Agricultural University, Guangzhou, China; ^3^ Institutes of Biomedical Sciences, Fudan University, Shanghai, China; ^4^ School of Life Sciences and Biopharmaceutics, Guangdong Pharmaceutical University, Guangzhou, China; ^5^ IMPART Investigator Team, Dalhousie Medicine, Saint John, NB, Canada; ^6^ Department of Biomedical Sciences, University of Guelph, Guelph, ON, Canada; ^7^ Shanghai Institute of Cardiovascular Diseases, Zhongshan Hospital, Fudan University, Shanghai, China; ^8^ Department of Biology, York University, Toronto, ON, Canada

**Keywords:** n-3 PUFA, IR injury, cardioprotection, APLNR, intracellular phosphorylation signaling

## Abstract

**Background:** While the protective effects of n-3 polyunsaturated fatty acids (PUFAs) on cardiac ischemia-reperfusion (IR) injury have been previously reported, limited data are available regarding how these fatty acids affect membrane receptors and their downstream signaling following IR injury. We aimed to identify potential receptors activated by n-3 PUFAs in IR hearts to understand the regulatory mechanisms of these receptors.

**Methods:** We used *fat-1* mice, which naturally have elevated levels of n-3 PUFAs, and C57BL/6J mice as a control group to create a myocardial IR injury model through Langendorff perfusion. We assessed the impact of endogenous n-3 PUFAs on left ventricular function, myocardial infarct size, myocardial apoptosis, and ATP production. RNA sequencing (RNA-seq) and bioinformatics analysis were conducted to identify molecular targets affected by n-3 PUFAs. Based on these analyses we then treated IR hearts of WT and *fat-1* mice with an antagonist (ML221) or an agonist (apelin-13) for the predicted receptor to assess cardiac contractile function and intracellular signaling pathways. An *in vitro* hypoxia-reoxygenation (HR) model was also used to confirm the effects of n-3 PUFAs on the examined intracellular signaling pathways.

**Results:** Endogenous n-3 PUFAs protected cardiac structure and function in post-IR hearts, and modulated phosphorylation patterns in the PI3K-AKT-mTOR signaling pathways. RNA-seq analysis revealed that n-3 PUFAs affected multiple biological processes as well as levels of the apelin receptor (APLNR). Consistent with a role for the PLNNR, ML221 synchronized the activation of the PI3K-AKT-mTOR signaling axis, suppressed the expression of PKCδ and phosphorylated p38α, upregulated PKCε expression, upregulated or restored the phosphorylation of myofilaments, and prevented myocardial injury and contractile dysfunction in WT IR hearts. By contrast, apelin-13 disrupted the PI3K-AKT-mTOR signaling axis in post-IR *fat-1* hearts. The phosphorylation signaling targeted by APLNR inhibition in post-IR *fat-1* hearts was also observed after treating HR cells with eicosatetraenoic acid (EPA).

**Conclusion:** Endogenous n-3 PUFAs protect against post-IR injury and preserve cardiac contractile function possibly through APLNR inhibition. This inhibition synchronizes the PI3K-AKT-mTOR axis, suppresses detrimental phosphorylation signaling, and restores or increases myofilament phosphorylation in post-IR hearts. The beneficial effects observed in *fat-1* transgenic mouse hearts can be attributed, at least in part, to elevated EPA levels. This study is the first to demonstrate that n-3 PUFAs protect hearts against IR injury through APLNR inhibition.

## Introduction

According to the American Heart Association (AHA) publication from the Global Burden of Disease Study 2020, the estimated incidence of ischemic heart disease is approximately 240 million (Tsao et al., 2022). Reperfusion therapies, including fibrinolytic therapy and percutaneous coronary intervention, primarily aim to restore blood flow to the ischemic myocardium, reducing morbidity and mortality in patients with acute myocardial infarction. However, reperfusion can also cause additional damage to cardiac tissues and disrupt normal function (Yellon and Hausenloy, 2007; Seeger et al., 2016).

Reperfusion injury can lead to heart failure (in approximately 25% of cases) and death (in approximately 10% of cases) in patients treated for acute myocardial infarction (Keeley et al., 2003; Heusch, 2020). It often induces oxidative stress, calcium overload, inflammation, fibrosis, and metabolic disorders (Carden and Granger, 2000; Yellon and Hausenloy, 2007). Various strategies, including treatment with n-3 polyunsaturated fatty acids (PUFA), have been proposed to prevent reperfusion injury.

A previous epidemiological study showed that Greenland Eskimos, with higher blood eicosatetraenoic acid (EPA) levels, had a lower incidence of myocardial infarction than did Danish participants (Dyerberg et al., 1978). More recent data have also demonstrated that n-3 PUFAs reduce the risk of coronary heart disease (Mozaffarian et al., 2005; Mozaffarian and Wu, 2011). The AHA’s scientific councils, including the Nutrition Committee of the Council on Lifestyle and Cardiometabolic Health and Epidemiology and Prevention, have recommended n-3 PUFA supplements for patients with recent myocardial infarction (Siscovick et al., 2017).

N-3 PUFAs, including alpha-linolenic acid (ALA), EPA, and docosahexaenoic acid (DHA), are primarily obtained from dietary sources, with a small amount of EPA/DHA converted from ALA. These fatty acids can be metabolized through enzymatic, non-enzymatic, or free radical-catalyzed pathways to produce oxylipins, exerting various functions (Gabbs et al., 2015; Nayeem, 2018). Their synthetic and metabolic properties enable multiple functions within cells. n-3 PUFAs act on metabolism, reduce oxidative stress, activate cellular membrane receptors, and alter cell membrane composition, all essential for maintaining cellular structural integrity, normal physiological function, and homeostasis (Calder, 2012).

Regarding intracellular regulation, evidence suggests that phosphorylation signaling plays a central role in n-3 PUFA-dependent cellular functions. For example, n-3 PUFAs regulate protein kinase C (PKC), mitogen-activated protein kinase (MAPK), and phosphatidylinositol 3-kinase (PI3K)/AKT to protect ischemic hearts (Engelbrecht et al., 2005; Szentandrássy et al., 2007; Siddiqui et al., 2008). For instance, in neonatal rat cardiomyocytes subjected to simulated ischemia-reperfusion (IR), EPA reduces apoptosis by activating ERK and dephosphorylating p38 MAPK (Engelbrecht et al., 2005). Our previous research also demonstrated that endogenous n-3 PUFAs significantly alter phosphorylation levels of intra- and extracellular proteins, protecting contractile function in pressure-overloaded mouse hearts (Li et al., 2022c).

However, while the link between n-3 PUFAs and intracellular phosphorylation signaling has been established, limited data exist on the membrane receptors involved. Fatty acid receptor 4 (FFA4) activation was found to regulate PPARγ-dependent sympathetic innervation in n-3 PUFA-protected infarcted rat hearts (Wang et al., 2022). Nevertheless, other membrane receptors may also play a role in the regulatory mechanisms of n-3 PUFAs. For example, in FFA4-knockout cardiomyocytes, the n-3 PUFA-derived oxylipin 18-HEPE prevents oxidative stress-induced cell death (Murphy et al., 2022). In this study, we employed bioinformatic analysis and molecular experimental tools to screen potential receptors activated by n-3 PUFAs in IR hearts, aiming to enhance our understanding of membrane receptor regulatory mechanisms.

## Materials and methods

### Animals and experimental design

Transgenic mice, kindly donated by Dr. Jing X. Kang from the Harvard Medical School (Boston, MA, United States ), were developed by expressing the n-3 fatty acid desaturase gene (*fat-1*) from *Caenorhabditis elegans*. This enzyme converts n-6 PUFAs into n-3 PUFAs, consequently elevating the endogenous levels of n-3 PUFAs in *fat-1* mice (Kang et al., 2004). The transgenic *fat-1* mice and wild-type (WT) C57BL/6J mice were bred and maintained in a specific pathogen-free (SPF) mouse facility accredited by the Association for Assessment and Accreditation of Laboratory Animal Care (AAALAC) and licensed by the Department of Science and Technology of Guangdong Province, China (SYXK[YUE] 2021-0122). Two primer pairs were used for genotyping the transgenic mice, namely, 5′-AGT​GGC​CTC​TTC​CAG​AAA​TG-3′ (forward) and 5′-TGC​GAC​TGT​GTC​TGA​TTT​CC-3′ (reverse) as well as 5′-CAC​AGT​GGG​TAA​CTG​GAT​GC-3′ (forward) and 5′-GCT​AAC​CAT​GTT​CAT​GCC​TTC-3` (reverse). The ambient temperature and humidity were 24°C ± 2°C and 40%–60%, respectively, with a 12-h light-dark cycle, and the mice had *ad libitum* access to water and provided a regular rodent diet. All animal experiments were conducted in accordance with the guidelines of the Institutional Animal Care and Use Committee of the Guangdong Laboratory Animals Monitoring Institute.

**FIGURE 1:**
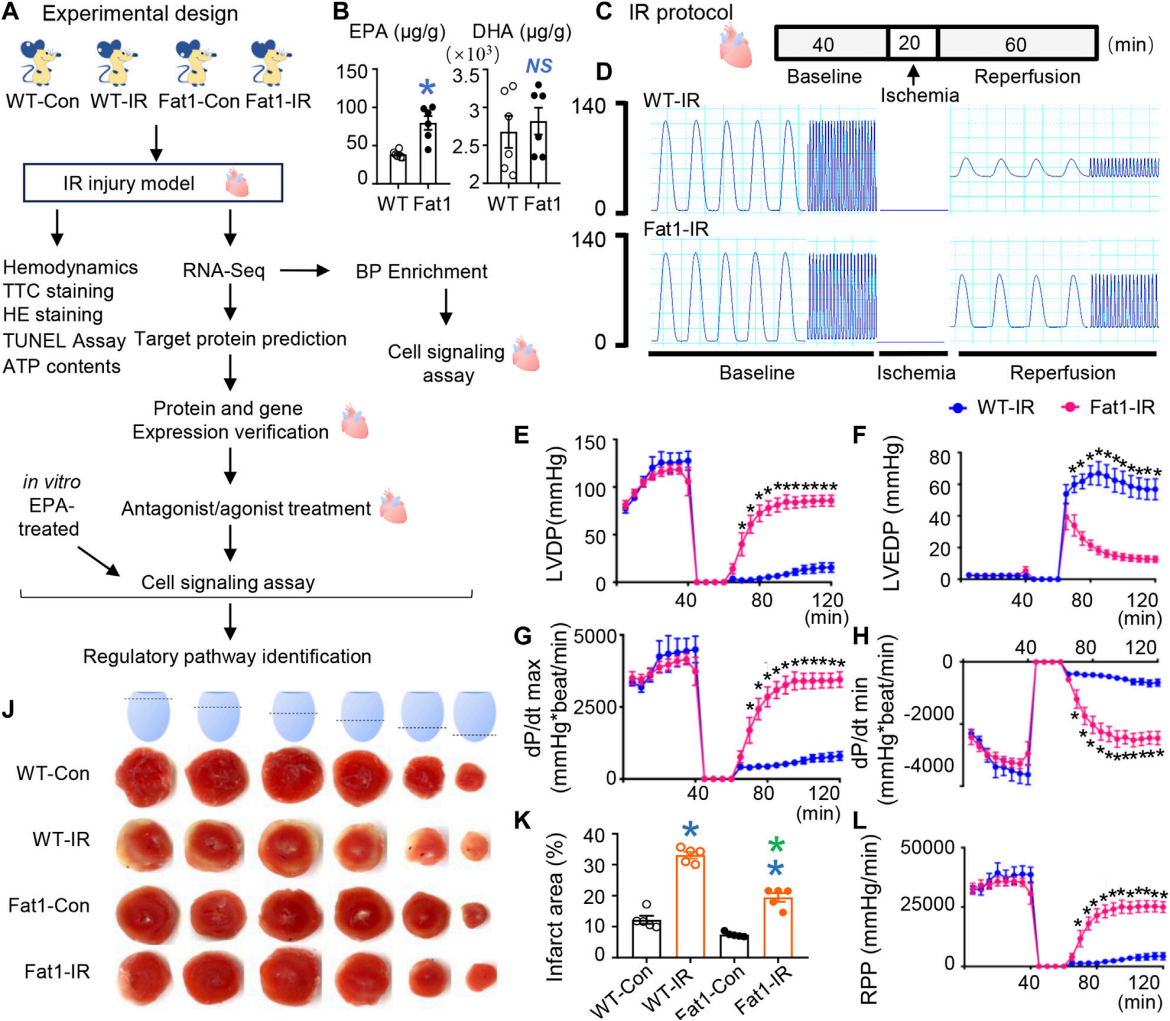
Effects of endogenous n-3 PUFAs on reperfusion injury. **(A)** Experimental design for this study. **(B)** Contents of n-3 PUFAs (EPA and DHA) in WT and *fat-1* mice hearts. *n* = 6 mice per group. **(C)** The WT-IR and Fat1-IR groups were subjected to 20 min of global ischemia, followed by 60 min of reperfusion. All hearts were perfused for 40 min for equilibration. The non-IR groups were perfused continuously for 120 min without global ischemia. **(D)** Representative traces of left ventricular pressure for WT and *fat-1* hearts during IR. Functional measurements (*n* = 8 mice) of WT-IR and Fat1-IR groups include left ventricular developed pressure (LVDP; **E**), left ventricular end-diastolic pressure (LVEDP; **F**), dP/dt(max) **(G)**, dP/dt(min) **(H)**, and rate pressure product (RPP; **I**). **(J)** Representative images of TTC staining in WT and *fat-1* hearts with or without reperfusion. **(K)** Quantitative analysis of the infarct area (*n* = 5 mice). WT-Con and Fat1-Con, WT or *fat-1* hearts not subjected to IR; WT-IR and Fat1-IR, WT or *fat-1* hearts subject to IR. Data are shown as the mean ± SEM; *p < 0.05 vs. WT (blue), WT-Con (blue) or WT-IR (green); NS, not significant compared with WT (blue).

WT and *fat-1* mice aged 3 months were subjected to the Langendorff heart preparation and perfusion. The cardiac contractility of the left ventricle was evaluated, followed by histological examination of the myocardium and RNA-seq analysis. The key gene targeted by n-3 PUFAs was identified, and the expression of this gene and encoding protein were verified. The antagonist (ML221) and agonist (apelin-13) of a specific gene-encoding protein were used to determine the associated signaling pathways. The experimental design is illustrated in Figure 1A.

### n-3 PUFA content in the heart

WT and *fat-1* mouse hearts were ground in liquid nitrogen. Methanol (150 μL), methyl tert-butyl ether (200 μL), and 36% of phosphoric acid (50 μL) were added to heart samples and centrifuged at 4°C and 12,000 × g to extract total lipids. The extracted fatty acids were transmethylated with 300 μL of 15% boron trifluoride-methanol was added and incubated at 60°C for 30 min. The resulting fatty acids were extracted with n-hexane and analyzed using a gas chromatography-mass spectrometry (GC-MS) platform (Agilent, 8890-5977B). Each integrated peak area representing a specific fatty acid was substituted into the standard curve equation to obtain the raw concentrations, which were further calibrated using the following equation:

$$\text{Fatty Acid Content}\left(\mu g/g\right)=c\times V3/1000\times V1/V2/m,$$

where c represents the concentration obtained by substituting the integrated peak area of the sample into the standard curve (μg/mL), V1 represents the volume of the sample extract (μL), V2 represents the volume of the collected supernatant (μL), V3 represents the reconstitution volume (μL), and m represents the sample mass (g).

### Establishment of the murine IR injury model

For determining the mechanisms underlying n-3 PUFA-induced cardioprotective effects on IR injury, Langendorff perfusion (Radnoti LLC, Covina, CA, United States) was performed to induce IR injury in mouse hearts, as previously described with modifications (Yang and Pyle, 2011; Li et al., 2020). Isolated hearts were perfused using a modified Krebs-Henseleit buffer solution and equilibrated with 95% O_2_/5% CO_2_; the perfusion pressure was set at 70 mmHg. A water-filled balloon connected to a pressure transducer and a PowerLab 16/35 system (ADInstruments, Dunedin, New Zealand) was used to record hemodynamic parameters, including left ventricular (LV) systolic and diastolic pressure, heart rate, and LV end-diastolic pressure (LVEDP). The LV developed pressure (LVDP), rate of pressure product (RPP), peak rate of contraction dP/dt(max), and peak rate of relaxation dP/dt(min) were analyzed using LabChart 7 software (ADInstruments).

For IR injury induction, isolated hearts were subjected to 40 min of baseline perfusion, followed by 20 min of no-flow global ischemia and 60 min of reperfusion. The 20-min antagonist/agonist treatments were conducted after 20 min of baseline perfusion. The control hearts were continuously perfused for 120 min. During reperfusion, heart tissues were fixed for subsequent hematoxylin-eosin (HE) staining, apoptotic analysis, RNA-seq analysis, gene expression verification, cell signaling assay, and detection of myofilament phosphorylation.

### Myocardial infarction assessment

Triphenyl tetrazolium chloride (TTC) staining of reperfused mouse hearts was performed as previously described (Yang and Pyle, 2012; Li et al., 2020) to evaluate the myocardial infarct size. The whole heart was cross-sectioned (2 mm thick) and incubated in 1% TTC solution (Sigma-Aldrich, St. Louis, MO, United States) at 37°C for 15 min. The red and white areas in the TTC-stained heart sections refer to the viable and infarcted tissues, respectively. The infarct size was measured using the ImageJ software (NIH, Bethesda, MD, United States).

### Histological examination

HE staining was conducted to assess the pathological changes in the reperfused mouse hearts, as previously described (Li et al., 2020; Li et al., 2022a). After embedding in paraffin, the hearts were sliced, dewaxed, and dehydrated, followed by staining with hematoxylin for 10 min and then with eosin for 25 s. The tissue sections were observed using an optical microscope (Leica DM 2000; Leica Microsystems, Wetzlar, Germany), and images of the tissues were recorded using the microscope imaging software (Leica Microsystems).

### TUNEL assay

TUNEL assay was performed to assess the apoptosis of myocardial cells in reperfused mouse hearts. Paraffin-embedded tissues were sectioned, dewaxed, dehydrated, and mounted on slides. The tissue sections were treated with proteinase K at 37°C for 30 min. After washing with phosphate-buffered saline (pH 7.4) solution thrice, 50 μL of TUNEL assay solution (#C1091; Beyotime, Shanghai, China) was added, and the slides were incubated at 37°C for 30 min in the dark, sealed with ProLong™ Gold Antifade Mountant with DAPI (#P36931; Thermo Fisher Scientific, Waltham, MA, United States), and observed under a fluorescence microscope (DMI3000 B, Leica Microsystems).

### ATP assay

ATP production was evaluated as previously described (Li et al., 2022c). Briefly, an ATP colorimetric assay kit (#A095-1-1; Jiancheng Biotechnology, Nanjing, China) was used to determine the amount of ATP in cardiac tissue homogenates, following the manufacturer’s instructions.

### RNA-seq analysis

Total RNA was extracted from the myocardium using TRIzol^®^ Reagent (Thermo Fisher Scientific). The concentration and integrity of the extracted RNA were assessed using RNA 6000 Nano Kit (Agilent Technologies, Santa Clara, CA, United States). The RNA-seq protocol used in this study has been previously published (Li et al., 2020; Li et al., 2022b; Li et al., 2022c). Briefly, sequencing libraries were generated using NEBNext^®^ Ultra™ RNA Library Prep Kit for Illumina^®^ (New England Biolabs, Ipswich, MA, United States). After adding the index codes, the samples were clustered with a cBot Cluster Generation System using TruSeq PE Cluster Kit v3-cBot-HS (Illumina, San Diego, CA, United States), and the HiSeq platform (Illumina) was used to sequence the libraries. The raw RNA-seq data were deposited in the NCBI Gene Expression Omnibus (GEO) database, the raw reads were preprocessed for quality control, and the clean reads were mapped to the reference transcriptome. The microarray GeneChip hybridization was carried out by Shanghai Applied Protein Technology Co., Ltd. (Shanghai, China).

The transcriptome data between groups were compared, and the fold change values between comparisons were log2-transformed. In this study, the cut-off for identifying differentially expressed genes (DEGs) was an adjusted *p* < 0.05. DEGs with fold change values >1.5 or <1/1.5 were considered upregulated or downregulated, respectively. A total of eight DEG datasets were generated, including the upregulated and downregulated comparisons for “WT-IR vs. WT-Con,” “Fat1-IR vs. Fat1-Con,” and “Fat1-IR vs WT-IR.” The web-accessible tool Metascape (https://metascape.org) was employed to enrich biological processes for two DEG datasets of “Fat1-IR vs. WT-IR”.

Furthermore, Venn diagram analysis was performed to predict the key target genes of n-3 PUFAs, with modifications as per our methods (Li et al., 2020). For predicting potential receptors involved in regulating endogenous n-3 PUFAs under IR conditions, the cut-off fold change value for DEG up- or downregulation comparisons between the two groups was set as > 2 or <2. To predict genes repressed/restored by n-3 PUFAs under IR conditions, we selected the genes that overlap the two datasets “Upregulated WT-IR vs WT-Con” and “Downregulated Fat1-IR vs WT-IR”, while excluding the genes that overlap with the dataset “Upregulated Fat1-IR vs Fat1-Con” and common DEGs between datasets. To predict genes activated/restored by n-3 PUFAs under IR conditions, we overlapped datasets “Downregulated WT-IR vs WT-Con” and “Upregulated Fat1-IR vs WT-IR”; genes obtained from this overlap were further filtered to exclude those overlapping with the dataset “Downregulated Fat1-IR vs Fat1-Con".

### Gene expression validation

Quantitative reverse transcription PCR (RT-qPCR) was performed to verify the expression of the identified DEGs. After total RNA extraction, TB Green Premix Ex Taq II (No. RR820; Takara Bio, Shiga, Japan) was used for RT-qPCR, following the manufacturer’s instructions. The reaction was run using a Real-Time PCR system (QuantStudio 5, ThermoFisher) as follows: initial denaturation at 95°C for 30 s, 40 cycles at 95°C for 5 s and 60°C for 34 s, and final extension at 72°C for 10 min. The transcript levels were normalized using β-actin as a reference. The forward and reverse primers used in this experiment were 5′-TCG​TGG​TGC​TTG​TAG​TGA​CC-3′ and 5′-ATG​CAG​GTG​CAG​TAC​GGA​AA-3′, respectively. The primers used for the reference gene (β-actin) were: 5′-GAT​ATC​GCT​GCG​CTG​GTC​G-3′ (forward) and 5′-CAT​TCC​CAC​CAT​CAC​ACC​CT-3′ (reverse).

### Cell signaling assay

Western blotting was used to determine the cell signaling pathways affected by n-3 PUFAs and identify those regulated by key genes in the n-3 PUFA-protected myocardia. The cytosolic fraction was extracted from cardiac tissues using a lysis buffer (#9803; Cell Signaling Technology, Danvers, MA, United States) containing 1% Triton X-100 and protease inhibitors (B14002; Bimake, Shanghai, China); phosphatase inhibitors (B15001; Bimake) was used in the samples for detecting phosphorylated proteins. Proteins (40 μg) were separated using 10% sodium dodecyl sulfate-polyacrylamide gel electrophoresis (SDS-PAGE) and transferred to polyvinylidene fluoride (PVDF) membranes. After blocking with 5% skim milk, the membranes were incubated with the primary antibodies (1:2000) overnight at 4°C and then with the secondary antibodies (1:5000). All antibodies were purchased from Cell Signaling Technology (Danvers, United States). The primary antibodies included antibodies against PKCδ (#9616), PKCε (#2683), p-p38α (Thr180/Tyr182; #4511), t-p38α (#9212), p-PI3K (Tyr458; #4228), t-PI3K (#4257), p-AKT (Ser473; #9271), t-AKT (#4685), p-mTOR (Ser2446; #2971), t-mTOR (#2972), APLNR (#35623), GAPDH (#2118), and the second antibody used was anti-rabbit (#7074). The bands were detected using Immobilon Western Chemiluminescent HRP Substrate (#WBKLS0500; Millipore, Burlington, MA, United States), and the band densities were analyzed using the ImageJ software (NIH).

### Detection of myofilament phosphorylation

The phosphorylation levels of the cardiac myofilaments were measured as previously described (Yang and Pyle, 2012; Zheng et al., 2021) The myofilaments (40 μg) were separated using 10% SDS-PAGE, and the gels were incubated with Pro-Q™ Diamond Phosphoprotein Gel Staining Solution (#P33300; Invitrogen Molecular Probes, Carlsbad, CA, United States) for 90 min, following the manufacturer’s instructions. The phosphorylated bands were visualized using a gel documentation system (Gel Doc™ XR+; Bio-Rad Laboratories, Hercules, CA, United States), and the band densities were quantified using the ImageJ software (NIH). The proteins were stained with Coomassie solution, and actin bands were used as loading controls.

### Treatment with APLNR antagonist and agonist

To investigate the effects of APLNR and its intracellular effectors on myocardial IR injury, we treated Landgendorff isolated hearts with ML221 (#HY-103254, MedChemExpress LLC, New Jersey, United States), an antagonist of APLNR, and Apelin-13, a potent and selective endogenous APLNR agonist. The first set of three experiment groups was designed for the inhibitor treatment: 1) WT-Con group, WT hearts were perfused for 120 min; 2) WT-IR group, WT hearts were balanced for 40 min, subjected to ischemia for 20 min, and then reperfused for 60 min; 3) WT-IR + M group, WT hearts were balanced for 20 min, then ML221 (50 nM) was added for 20 min, followed by ischemia for 20 min and reperfusion for 60 min. After reperfusion, the hearts were subjected to TTC staining, Western blot analysis, and detection of myofilament phosphorylation.

The second set of three experiment groups was designed for the agonist treatment. 1) Fat1-Con group: *fat-1* mouse hearts were perfused for 120 min; 2) Fat1-IR group, *fat-1* mouse hearts were perfused for 40 min and then subjected to ischemia for 20 min and reperfusion for 60 min; 3) Fat1-IR + A group: *fat-1* hearts were perfused for 40 min, then treated with apelin-13 (50 nM) for 20 min, followed by ischemia for 20 min and reperfusion for 60 min. After reperfusion, all hearts were subjected to Western blot analysis.

### EPA treatment

H9C2 cells (iCell-r012) were employed to assess the expression of APLNR and intracellular phosphorylation signaling components following hypoxia and reoxygenation (principal components of myocardial IR). The H9C2 cell line is commonly used to investigate the molecular mechanisms of ischemia, IR injury, and IR-preconditioning. The cells were cultured in Dulbecco’s modified Eagle medium (DMEM; Gibco, C11995500BT) containing 10% fetal bovine serum (FBS; Gibco, 10099-141) and 1% penicillin-streptomycin diabody (Yeasen, 60162ES76) and incubated at 37°C and 5% CO_2_. The cells were divided into four groups, namely, Con, hypoxia-reoxygenation (HR), Con + EPA, and HR + EPA.

Treatment was initiated when the cells reached 90% confluency. The regular culture medium was replaced with a sugar-free, serum-free DMEM (Gibco, 11966025). The cells of the IR and IR + EPA groups were placed in a hypoxic apparatus at 37°C, 94% N_2_, 5% CO_2_, and 1% O_2_. After 24 h of hypoxia, the normal medium containing 10% FBS was replaced. The hypoxia and reoxygenation protocol was used to simulate *in vivo* IR conditions. In the EPA treatment groups (Con + EPA and HR + EPA), 100 nM EPA (MCE, HY-B0660) was added, and the cells were incubated at 37°C and 5% CO_2_ for 6 h. Cells in the Con and Con + EPA groups were cultured continuously for 30 h at 37°C and 5% CO_2_ without hypoxia. The Con + EPA and HR + EPA groups were treated with 100 nM EPA at the same time. Cardiomyocytes were collected following treatment, and protein analysis using Western blotting was conducted.

### Statistical analysis

Data are presented as means ± SEM. The raw data from each group were analyzed using GraphPad Prism 8.0 software (GraphPad, San Diego, CA, United States) to determine significant differences between groups. For comparison between two groups, an unpaired *t-*test was used, while for comparison between multiple groups, a one-way analysis of variance (ANOVA) was performed, followed by Tukey’s multiple comparison test. Statistical significance was set at *p* < 0.05.

## Results

### Elevated endogenous n-3 PUFA levels preserve contractile function and prevent myocardial damage

The EPA levels in *fat-1* hearts were approximately two times higher than those in WT hearts. Additionally, there were no differences in DHA concentrations between the hearts of WT and *fat-1* mice, as shown in Figure 1B. The baseline cardiac function parameters, including dP/dt(max), LVDP, LVEDP, and RPP, did not exhibit significant differences between WT and *fat-1* mice, as indicated in Figures 1C–I. However, after 20 min of ischemia, dP/dt(max), LVDP, and RPP values were notably higher, while LVEDP was significantly lower in *fat-1* mice than those in WT mice at each time point during the 60-min reperfusion, as demonstrated in Figures 1D–I. These improvements in contractility were strongly associated with a considerable reduction in infarct size in the hearts of Fat1-IR mice, as shown in Figures 1J, K. These findings suggest that the elevated endogenous levels of n-3 PUFAs provide significant benefits to reperfused myocardia, resulting in improved systolic and diastolic function and reduced myocardial damage.

### n-3 PUFAs target multiple biological processes in reperfused mouse hearts

**FIGURE 2:**
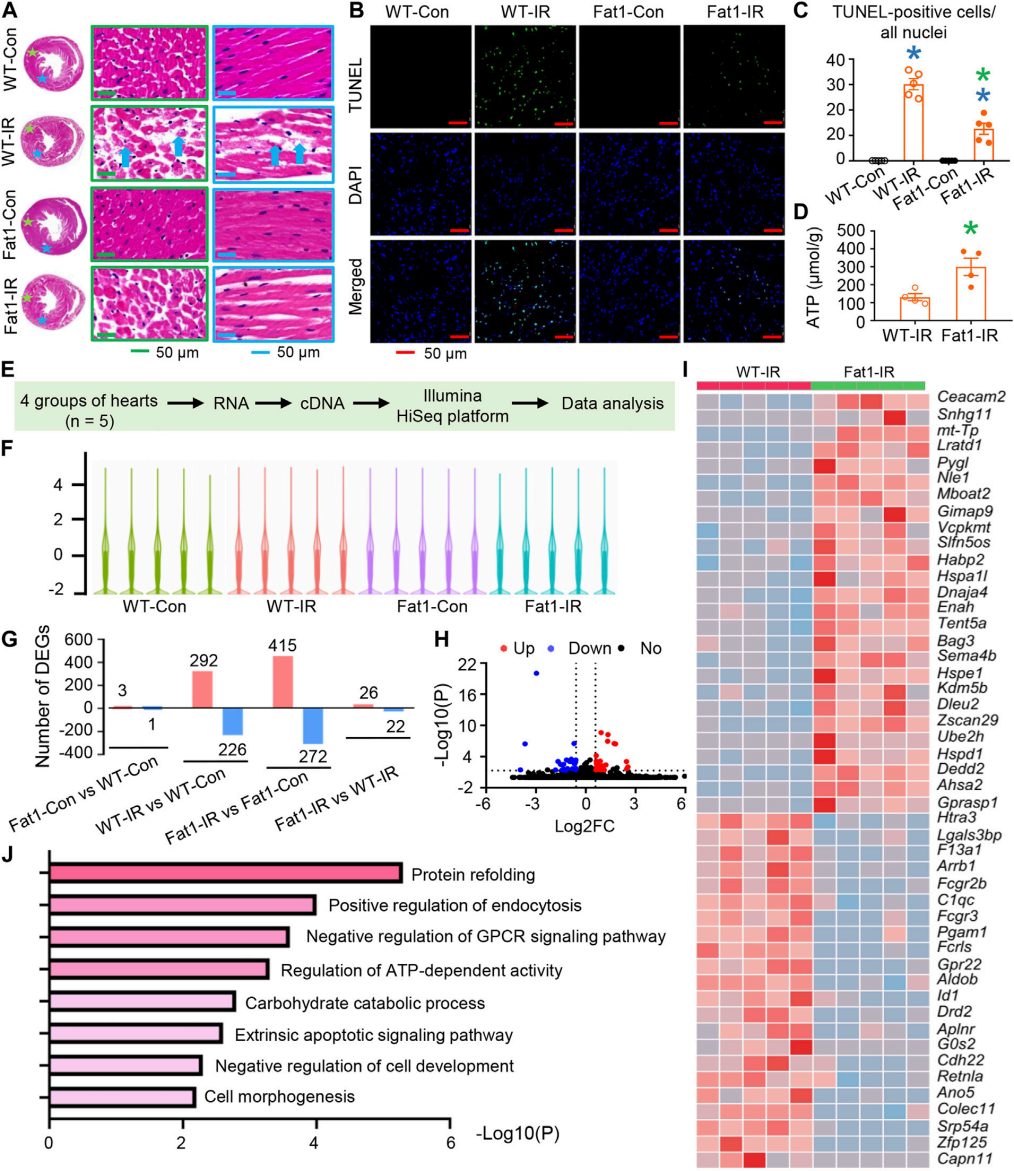
Cell survival and gene expression profiles of post-IR WT and *fat-1* hearts. **(A)** Representative images of hematoxylin-eosin (HE) staining in WT and *fat-1* hearts, showing lost or ruptured myofibers (blue arrows) in WT-IR hearts. Regions with stars correspond to images with boxes of the same color. **(B)** Representative images of TUNEL assay in WT and *fat-1* hearts with or without reperfusion. Green, TUNEL-positive cells; blue, nuclei. **(C)** Quantitative analysis of TUNEL-positive cells in each group. *n* = 5 mice per group. **(D)** ATP production in WT-IR and Fat1-IR hearts. *n* = 4 mice. **(E)** Flow diagram for profiling gene expression of mouse hearts with or without ischemia-reperfusion (IR). *n* = 5 mice. **(F)** The distributions of the total mRNAs in each sample. **(G)** Counts of up- and downregulated differentially expressed genes (DEGs) in each comparison. **(H)** Volcano plot and **(I)** heatmap for DEGs between WT-IR group and Fat-IR group. **(J)** Gene set enrichment analysis (GESA) for WT-IR vs. Fat1-IR. WT-Con and Fat1-Con, WT or *fat-1* hearts not subjected to IR; WT-IR and Fat1-IR, WT or *fat-1* hearts subject to IR. Data are shown as the mean ± SEM; *p < 0.05 vs. WT-Con (blue) or WT-IR (green).

Microscope assessments revealed lost or ruptured fibers in WT-IR mouse myocardia, while Fat1-IR mouse myocardia showed no severe fiber ruptures (Figure 2A). Furthermore, there were fewer focal apoptotic cells in the Fat1-IR mouse hearts than in the WT-IR mouse hearts (Figures 2B, C). Additionally, following IR, *fat-1* hearts exhibited higher ATP production than did WT hearts (Figure 2D). These results suggest that elevated endogenous n-3 PUFAs protect cell structure, promote cell survival, and enhance metabolism in reperfused myocardium.

We also conducted gene expression profiling in post-IR mouse hearts. Transcriptomic analysis revealed alterations in the distribution of total mRNAs in each sample (Figures 2E–G). Specifically, in WT hearts, 292 and 226 differentially expressed genes (DEGs) were upregulated and downregulated following IR, respectively. In contrast, in *fat-1* hearts, 415 DEGs were upregulated, and 272 were downregulated post-IR (Figure 2G). Furthermore, only 3 genes were upregulated, and 1 was downregulated in *fat-1* hearts compared with those in WT hearts. However, following IR, 26 DEGs were upregulated, and 22 were downregulated in *fat-1* hearts compared with those in WT hearts. DEGs were listed in Supplementary Table S1.

DEGs between WT and *fat-1* mice subjected to IR were shown in The Volcano plot and heatmap (Figures 2H, I). Gene set enrichment analysis (GSEA) predicted various biological processes influenced by n-3 PUFAs in the hearts subjected to IR (Figure 2J). These processes encompassed morphological and functional regulation, including protein refolding, negative regulation of cell development, cell morphogenesis, and negative regulation of G protein-coupled receptor signaling pathways. Additionally, survival regulation included the extrinsic apoptotic signaling pathway, while metabolic regulation encompassed the regulation of ATP-dependent activity and carbohydrate catabolic processes. These findings align with our functional analyses, ATP production measurement and observations related to cell survival.

### Endogenous n-3 PUFAs regulate contractile-associated protein kinases and synchronize PI3K-AKT-mTOR phosphorylation in post-IR myocardium

**FIGURE 3:**
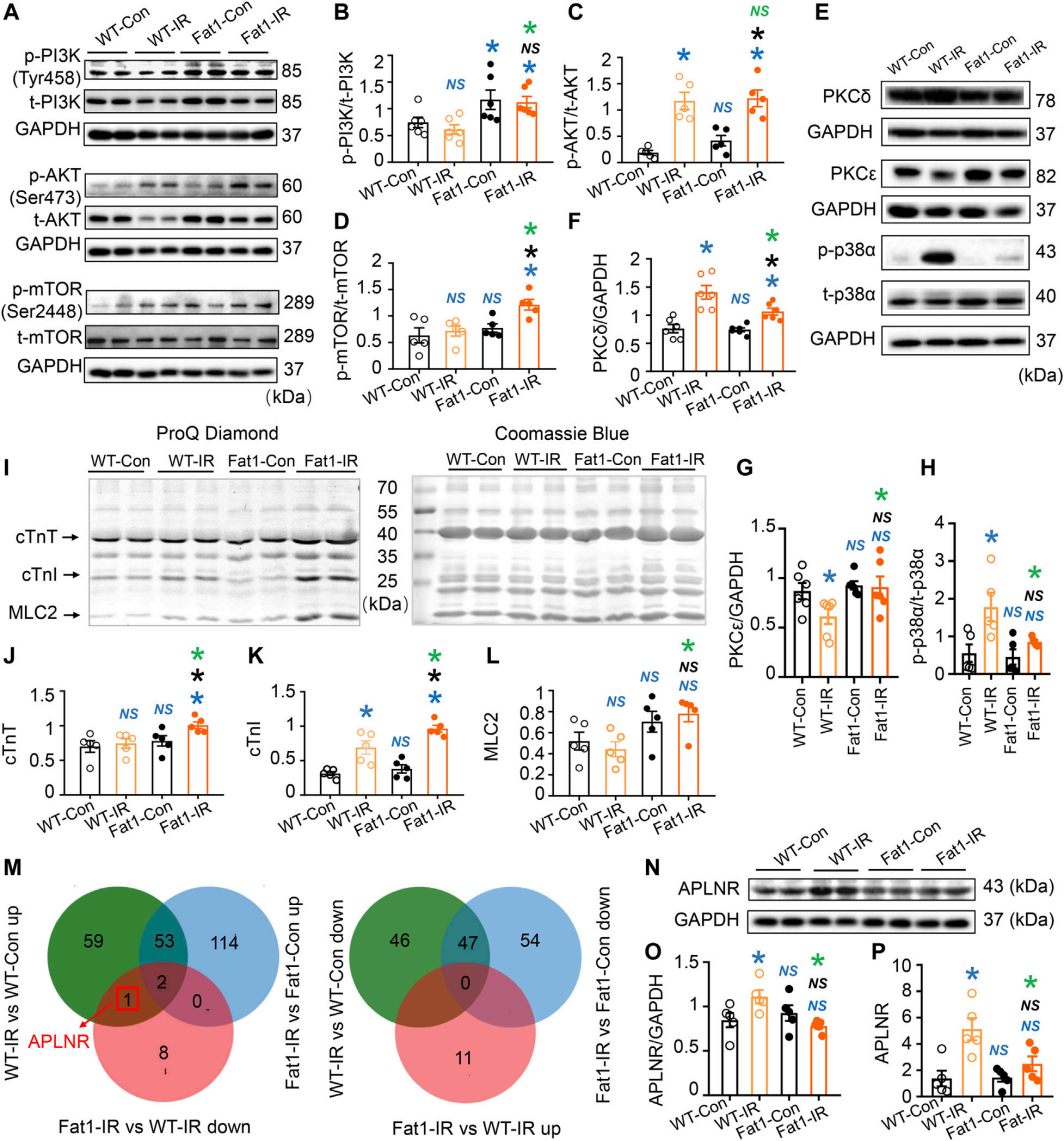
Expression of protein kinases, phosphorylation of myofilaments, and prediction of novel regulatory mechanisms induced by n-3 PUFAs in IR myocardia. **(A–D)** Representative images and quantitative analysis of expression of phosphorylated (p-) and total (t-) PI3K, AKT, and mTOR. **(E–H)** Representative images and quantitative analysis of PKCδ, PKCε, p-p38α, t-p38α, and GAPDH. *n* = 5-6 mice per group. (**I**) ProQ phosphorylation staining and Coomassie staining of cardiac myofilaments in the heats (WT and *fat-1*) with or without IR. **(J–L)** Quantitative analysis of phosphorylation levels of cTnT, cTnI, and MLC2. *n* = 5 mice per group. **(M)** Venn diagram of the differentially expressed genes (DEGs) in two comparison groups. **(N,O)** Representative images and the quantitative analysis of protein expression of APLNR in the hearts (WT and *fat-1*) with or without IR. *n* = 5 mice per group. **(P)** Quantitative analysis of gene expression of APLNR in non-IR and IR hearts. *n* = 5 mice per group. Data are shown as the mean ± SEM; *p < 0.05 vs. WT-Con (blue), Fat1-Con (black) or WT-IR (green); NS, not significant compared with WT-Con (blue), Fat1-Con (black) or WT-IR (green).

We investigated the potential involvement of intracellular phosphorylation signaling pathways following gene set enrichment analysis. Initially, we examined the PI3K-AKT-mTOR signaling pathway (Figures 3A–D), a well-known regulator of cell metabolism, proliferation, and survival. In post-IR WT hearts, the phosphorylation levels of PI3K, AKT, and mTOR remained unchanged, were upregulated, and remained unchanged, respectively. However, all these signaling molecules were upregulated in Fat1-IR mice compared with WT-Con mice. This suggests that these signaling molecules became uncoupled in response to IR while elevated endogenous n-3 PUFAs synchronized the activation of this signaling axis.

Next, we assessed the expression of PKCδ, PKCε, and p-p38α, which are associated with myocardial contractility in reperfused hearts (Yang and Pyle, 2012; Li et al., 2020). We found that the protein expression of PKCε was downregulated, while the expression of PKCδ and p-p38α was upregulated in WT-IR myocardia. Compared with WT-IR mice, Fat1-IR mice have higher expression levels of PKCε but lower expression levels of PKCδ and p-p38α (Figures 3E–H).

Furthermore, we evaluated the phosphorylation levels of cardiac myofilaments after observing their involvement in G protein-coupled receptor (GPCR)-activated protein kinase signal transduction. We found that IR increased cardiac troponin I (cTnI) phosphorylation levels in WT mice. However, under IR conditions, *fat-1* mice had higher phosphorylation levels of cTnT, cTnI, and myosin light chain 2 (MLC2) than WT mice (Figures 3I–L). These findings suggest that myofilament phosphorylation represents a potential target of n-3 PUFAs.

### APLNR inhibition associated with improved cardiac function

We explored the regulatory mechanisms of endogenous n-3 PUFAs involved in preventing reperfusion injury. Using Venn diagram analysis (Figure 3M), we found that the gene encoding apelin receptor (APLNR, also known as APJ) is upregulated by IR, but this upregulation is inhibited by n-3 PUFAs under IR conditions. Next, the expression levels of APLNR protein and gene were verified using Western blotting (Figures 3N, O) and RT-qPCR (Figure 3P), respectively, and the results were consistent with the transcriptomics data. This suggests that APLNR is likely involved in the cardioprotective effects of n-3 PUFAs.

**FIGURE 4:**
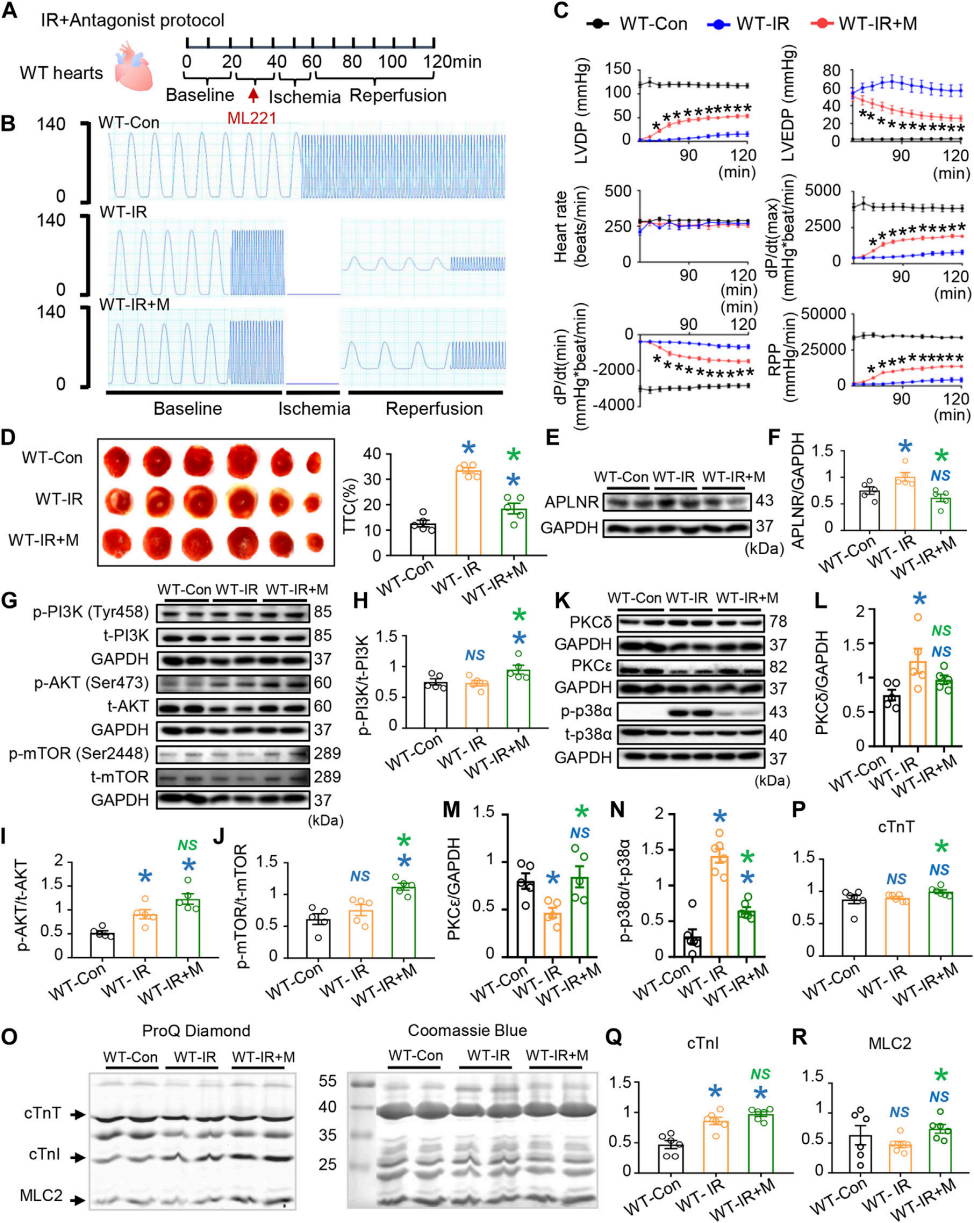
Effects of apelin receptor (APLNR) antagonist (ML221) on post-IR WT hearts. **(A)** Antagonist treatment protocol. “M” indicates APLNR antagonist ML221. **(B)** Representative traces of left ventricular pressure for the WT-Con, WT-IR, and WT-IR + M groups. **(C)** Functional measurements include heart rate, left ventricular developed pressure (LVDP), left ventricular end-diastolic pressure (LVEDP), dP/dt(max), dP/dt(min), and rate pressure product (RPP). *n* = 7–8 mice per group. **(D)** Representative images and quantitative analysis of TTC staining in hearts of WT-Con, WT-IR, and WT-IR + M groups. n = 5 mice per group. **(E,F)** Protein expression of APLNR in IR myocardia after treatment with ML221. **(G–J)** Representative images and quantitative analysis of expression of phosphorylated (p-) and total (t-) PI3K **(H)**, AKT **(I)**, and mTOR **(J)**. *n* = 5 mice per group. **(K–N)** Representative images and quantitative analysis of expression of PKCδ **(L)**, PKCε **(M)**, and phosphorylated (p-) and total (t-) p38α **(N)**. *n* = 5–6 mice per group. **(O)** Representative images of ProQ phosphorylation staining and Coomassie staining of cardiac myofilaments. Quantitative analysis of phosphorylation levels of cTnT **(P)**, cTnI **(Q)**, and **(R)**. *n* = 6 mice per group. Data are shown as the mean ± SEM; *p < 0.05 vs. WT-Con (blue) or WT-IR (green); NS, not significant compared with WT-Con (blue) or WT-IR (green).

Furthermore, we observed that treatment with APLNR antagonist partially restored the LVDP, dP/dt(max), and RPP and suppressed the increase in LVEDP and dP/dt(min) in WT-IR hearts (Figures 4A–C). Additionally, TTC staining results showed that the APLNR antagonist reduced the infarction area in reperfused mouse hearts (Figure 4D). Therefore, these results indicate that APLNR inhibition improves cardiac contraction and relaxation and protects cardiac structure, consistent with those of endogenous n-3 PUFAs.

Subsequently, we investigated APLNR-mediated signaling activation in reperfused mouse hearts. Firstly, we verified that APLNR expression was upregulated in WT hearts post-IR, an effect suppressed by ML221 (Figures 4E, F). Next, ML221 treatment increased the phosphorylation of PI3K and mTOR and maintained AKT phosphorylation in WT hearts post-IR (Figures 4G–J). These results suggest that APLNR inhibition synchronized the activation of PI3K, AKT, and mTOR in post-IR WT hearts, compared with that in untreated hearts, which showed AKT activation alone. This synchronized activation of the PI3K-AKT and mTOR pathways resembled that observed in post-IR hearts protected by endogenous PUFAs (Figures 3A–D).

Additionally, ML221 treatment suppressed the increased phosphorylation of p38α in post-IR WT hearts (Figures 4K, N). After IR, PKCδ expression increased but PKCε expression decreased in WT hearts (Figures 4K–M). Similar to the APLNR inhibition induced by endogenous n-3 PUFAs in post-IR hearts, ML221 restored the expression of PKCε and prevented the expression of PKCδ and p-p38α after IR (Figures 3F–H).

The phosphorylation of cardiac myofilaments (cTnT, cTnI, and MLC2) was also assessed to identify the targets of APLNR inhibition in post-IR WT hearts (Figures 4O–R). Similar to the findings in *fat-1* IR hearts with APLNR inhibition, ML221 increased cTnT phosphorylation in post-IR WT hearts but did not further increase cTnI phosphorylation (Figures 4O–Q; Figures 3I–K). Endogenous n-3 PUFAs (APLNR inhibited) and the APLNR antagonist increased MLC2 phosphorylation in post-IR *fat-1* and post-WT hearts, respectively (Figure 4R; Figure 3L).

In conclusion, APLNR inhibition benefits cardiac contraction and relaxation, synchronizes phosphorylation of the PI3K-AKT-mTOR axis, and regulates contractile-associated protein kinases and myofilament phosphorylation.

### APLNR agonist abolishes the protective effects provided by endogenous n-3 PUFAs

**FIGURE 5:**
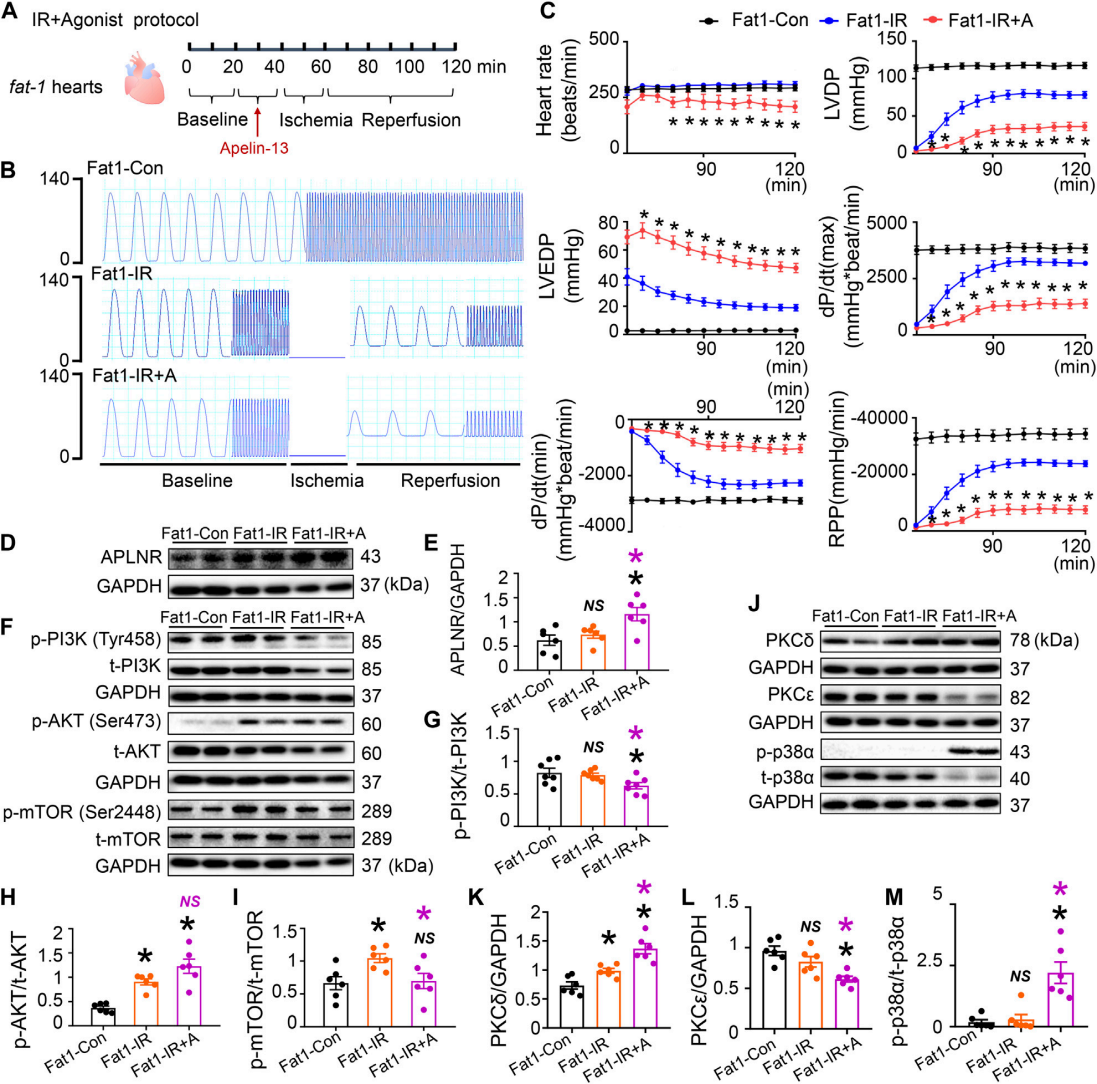
Role of apelin receptor (APLNR) agonist (Apelin-13) in the reperfused *fat-1* heart. **(A)** Agonist treatment protocol. Fat1-IR hearts were subjected to 20 min of global ischemia, followed by 20 min of apelin-13 treatment and 20 min of ischemia. Both the Fat1-IR and Fat1-IR + A groups were reperfused for 60 min. The non-IR groups were not subject to global ischemia. **(B)** Representative traces of left ventricular pressure for the Fat1-Con, Fat1-IR, and Fat1-IR + A groups. **(C)** Functional measurements include heart rate, left ventricular developed pressure (LVDP), left ventricular end-diastolic pressure (LVEDP), dP/dt(max), dP/dt(min), and rate pressure product (RPP). *n* = 7–8 mice per group. **(D,E)** Protein expression of APLNR in IR myocardia after treatment with apelin-13. **(F–I)** Representative images and quantitative analysis of expression of phosphorylated and total PI3K **(G)**, AKT **(H)**, and mTOR **(I)**. *n* = 6 mice per group. **(J–M)** Representative images and quantitative analysis of expression of PKCδ **(K)**, PKCε **(L)**, and phosphorylated (p-) and total (t-) p38α **(M)**. n = 6 mice per group. Data are shown as the mean ± SEM; *p < 0.05 vs Fat1-Con (black) or Fat1-IR (purple); NS, not significant compared with Fat1-Con (black) or Fat1-IR (purple).

Treatment with the APLNR agonist (apelin-13) significantly reversed the heart rate, LVDP, LVEDP, dP/dt(max), dP/dt(min), and RPP improvements brought about by endogenous n-3 PUFAs in post-IR *fat-1* hearts (Figures 5A–C). These functional results confirm that endogenous n-3 PUFAs protect post-IR hearts through APLNR inhibition. Apelin-13 increased APLNR expression in the post-IR *fat-1* myocardia (Figures 5D, E). Apelin-13 reduced PI3K and mTOR phosphorylation levels, while AKT phosphorylation levels remained unchanged in the post-IR *fat-1* myocardia (Figures 5F–I). Furthermore, apelin-13 increased PKCδ expression and p-p38α phosphorylation while decreasing PKCε expression in post-IR *fat-1* hearts (Figures 5J–M). Therefore, the protein expression results suggest that the upregulation of APLNR can disrupt the endogenous n-3 PUFAs-activated PI3K-AKT-mTOR signaling axis and mediate contraction-related signaling pathways, including PKC and p38 MAPK pathways.

### EPA is a key player in APLNR inhibition-induced cardioprotection under ischemic condition

**FIGURE 6:**
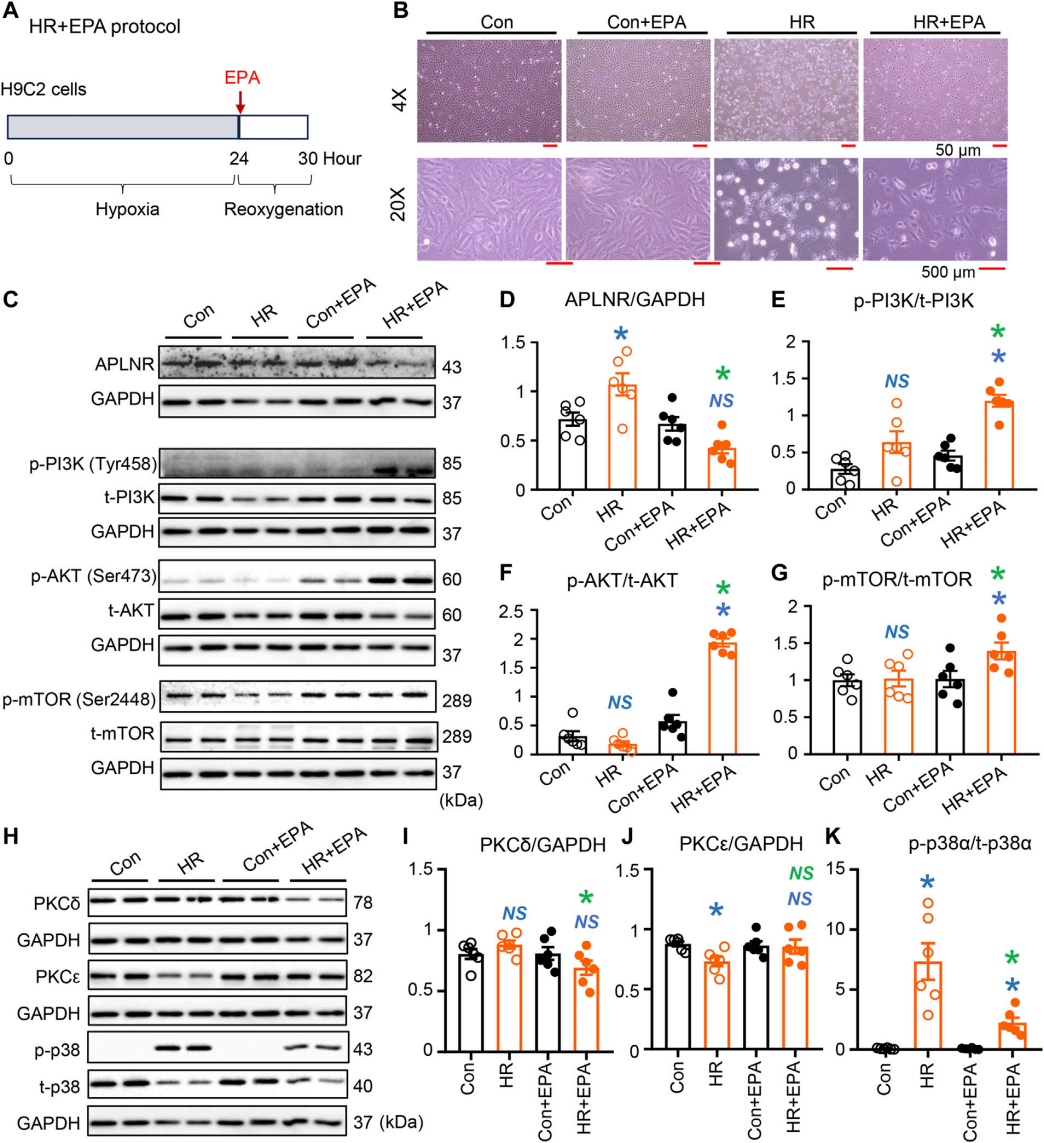
Expression of intracellular protein kinases in EPA-treated H9C2 cells under simulated IR conditions. **(A)** EPA treatment protocol. Cells were deprived of oxygen for 24 h, then EPA was added and reoxygenated for 6 h. EPA treatment improved morphology of the cell subjected to HR **(B)**. Protein expression of APLNR was inhibited in hypoxia-reoxygenation (HR) cells after treatment with EPA **(C,D)**. EPA treatment alters the activation of the PI3K-AKT-mTOR signaling axis, as displayed in the representative images **(C)** and quantitative analysis of expression of phosphorylated and total PI3K **(E)**, AKT **(F)**, and mTOR **(G)**. *n* = 6 mice per group. **(H–K)** Representative images and quantitative analysis of expression of PKCδ **(I)**, PKCε **(J)**, and phosphorylated (p-) and total (t-) p38α **(K)**. *n* = 6 mice per group. Data are shown as the mean ± SEM; *p < 0.05 vs. Con (blue) or HR (green); NS, not significant compared with Con (blue) or HR (green).

*In vitro* experiments conducted on EPA-treated H9C2 cells under hypoxia-reoxygenation (simulated IR conditions) revealed alterations in cell morphology and components of intracellular signaling pathways (Figure 6A). HR significantly damaged the organization of H9C2 cells; however, EPA partially maintained the morphology of cells subjected to HR (Figure 6B). Protein expression of APLNR was inhibited by EPA treatment in HR cells (Figures 6C, D), which aligns with the findings in post-IR *fat-1* hearts with elevated n-3 PUFA levels (mainly EPA; Figures 3N, O). EPA treatment synchronized the phosphorylation of the PI3K-AKT-mTOR signaling axis in post-HR cells (Figures 6C, E–G), mirroring the findings in post-IR *fat-1* hearts that also showed synchronization of this signaling axis (Figures 3A–D). Furthermore, the expression of PKCδ and p-p38α was decreased by EPA treatment in HR cells (Figures 6H, I, K), consistent with the effects observed in the post-IR *fat-1* mouse myocardia (Figures 3F, H). Additionally, EPA did not alter PKCε expression (Figure 6J). These results suggest that EPA is crucial in mediating APLNR inhibition-dependent cardioprotective signaling under ischemic conditions.

## Discussion

**FIGURE 7:**
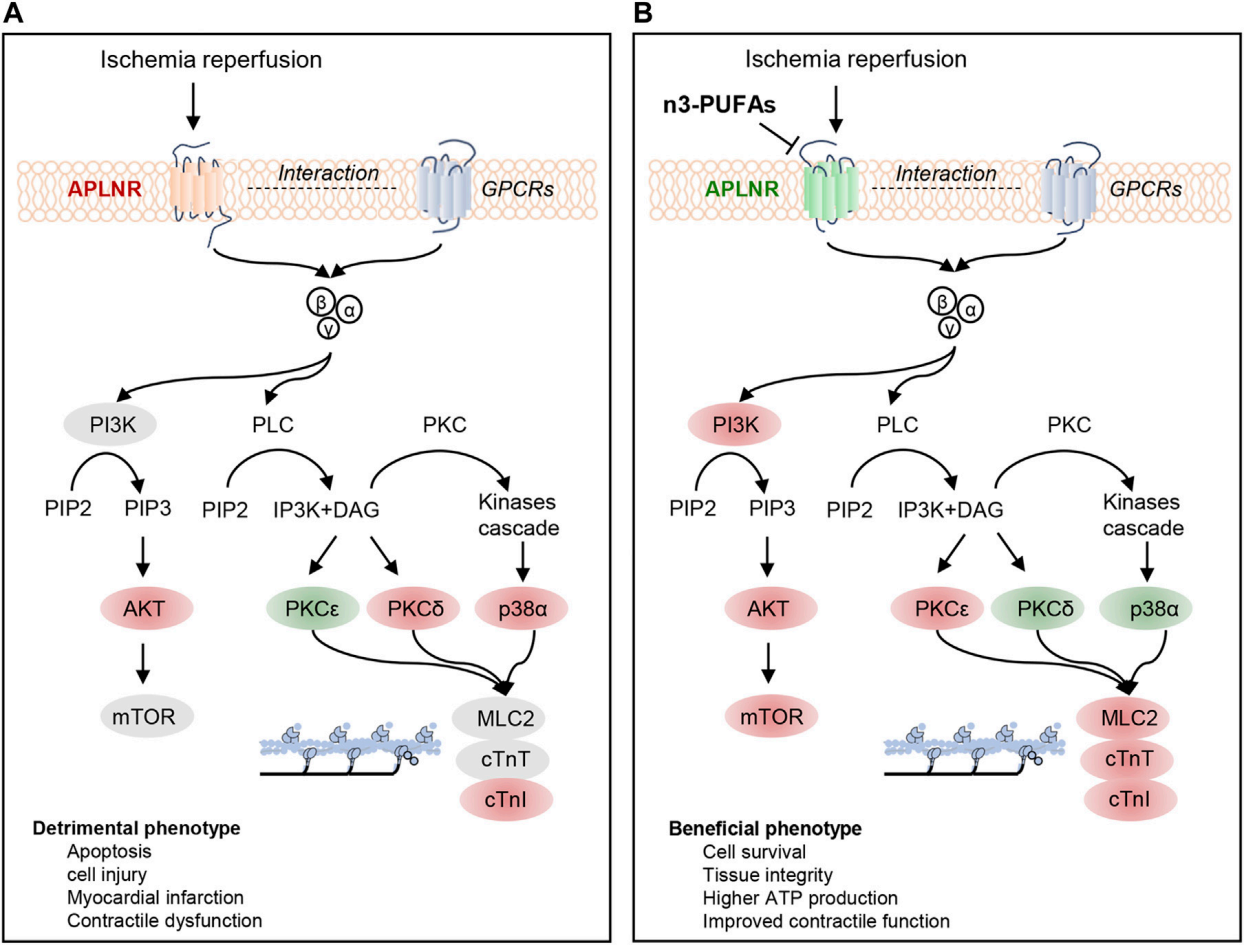
Regulatory mechanisms of APLNR inhibition in ischemia-reperfused hearts protected by n-3 PUFAs. **(A)** Ischemia-reperfusion (IR) activates the apelin receptor (APLNR) and is associated with the phosphorylation of partially PI3K-AKT-mTOR signaling axis, increased expression of p-p38α and PKCδ, decreased expression of PKCε, and elevated phosphorylation levels of cTnI. **(B)** n3-PUFAs inhibit the expression of APLNR, leading to synchronized activation of the PI3K-AKT-mTOR signaling axis, decreased expression of PKCδ and p-p38α, and increased expression of PKCε. APLNR inhibition induced by n-3 PUFAs leads to beneficial phenotypes. Red indicates an increase in protein expression or phosphorylation compared with WT-Con or WT-IR, while green indicates a decrease in protein expression or phosphorylation compared with those in WT-Con or WT-IR.

In this study, we investigated the cardioprotective effects and underlying mechanisms of endogenous n-3 PUFAs in mice subjected to IR. Our findings revealed that endogenous n-3 PUFAs in the myocardium preserve contractile function by modulating intracellular signaling pathways in Langendorff-prepared IR hearts. Furthermore, we identified and confirmed APLNR as a mediator of n-3 PUFAs-dependent intracellular signaling. Specifically, inhibiting APLNR led to the synchronized activation of the PI3K-AKT-mTOR signaling pathway, favorable changes in the expression profiles of PKC and p-p38α (with suppression of PKCδ and p-p38α and upregulation of PKCε), and the maintenance or enhancement of myofilament phosphorylation (cTnT, cTnI, MLC2) in the hearts protected by n-3 PUFAs during IR (Figure 7). Consequently, inhibiting this receptor reduced the myocardial infarcted area and improved the contraction and relaxation of the reperfused hearts. This study identifies novel targets of n-3 PUFAs in the myocardium following IR and provides insights into new therapeutic strategies for mitigating IR injury.

### APLNR inhibition induced by endogenous n-3 PUFAs is a novel cardioprotective mechanism after IR

APLNR is a GPCR with seven transmembrane domains, first reported in 1993 (O'Dowd et al., 1993). APLNR is found in various organs and tissues, including the heart (Medhurst et al., 2003). This receptor has two known endogenous ligands, apelin and Elabela/Toddler (Tatemoto et al., 1998; Chng et al., 2013; Pauli et al., 2014). Apelin exerts dose-dependent positive inotropy and acute cardiovascular effects in humans (Szokodi et al., 2002; Japp et al., 2010). Infusion of apelin increased stroke volume in ischemic cardiomyopathic rat hearts induced by left anterior descending coronary artery ligation (Berry et al., 2004). Apelin regulates G proteins in fatty acid metabolism and interacts with intracellular signaling pathways including PI3K/AKT, MAPK, PKC pathways(Yue et al., 2011; Vitale et al., 2022). Furthermore, APLNR may signal in the absence of endogenous ligands. This receptor formed heterodimer with angiotensin II receptor, κ-opioid receptor and other GPCRs to modulate G protein mediated signal transduction(Chun et al., 2008; Li et al., 2012; O'Carroll et al., 2013). APLNR also acted as a mechanosensory to induce cardiomyocyte stretch in an apelin-independent manner (Scimia et al., 2012).

APLNR is crucial for heart development, cardiac function, energy metabolism, and cell differentiation (Pitkin et al., 2010; Deshwar et al., 2016). Under pathological conditions, accumulating evidence suggests that the apelin/APLNR system has multiple roles (Lu et al., 2017; de Oliveira et al., 2022). In terms of beneficial effects, apelin improved cardiac function in mice with post-myocardial infarction heart failure dilated cardiomyopathy (Berry et al., 2004; El Mathari et al., 2021). Furthermore, apelin also reduced blood pressure in DOCA salt-induced hypertensive rats (Akcılar et al., 2013). However, this receptor may become uncoupled from the activation of apelin in pathological heart conditions. For instance, apelin mRNA levels in the left ventricles were increased approximately 4.7- and 3.3-fold in coronary heart disease-induced and idiopathic dilated cardiomyopathy-induced chronic heart failure, respectively, whereas APLNR mRNA was downregulated in the left ventricle of coronary heart disease-induced heart failure (Földes et al., 2003). Furthermore, mouse hearts knocking out APLNR are resistant to pressure overload-induced dilated heart failure (Scimia et al., 2012).

We observed that the mRNA and protein expression of APLNR was upregulated in response to IR or HR conditions in *ex vivo* hearts or *in vitro* H9C2 cells, respectively. These findings are consistent with the increase in APLNR expression in reperfused hearts (approximately 15 min after ischemia) reported by Rastaldo et al. (2011). Furthermore, our study showed that the inhibition of this receptor, either by elevated endogenous n-3 PUFAs or ML221, improved contraction and relaxation in the post-IR mouse hearts. To the best of our knowledge, this is the first study to demonstrate that APLNR inhibition protects hearts against IR injury. Additionally, we found that APLNR inhibition reduced myocardial infarction. Since cardiac infarction is a causative factor in cardiac remodeling in ischemic hearts (Sutton and Sharpe, 2000), APLNR inhibition may enable IR hearts to resist chronic maladaptive remodeling, as observed in pressure overload hearts by Scimia et al. (2012).

### n-3 PUFA protects the heart by modulating the APLNR inhibition-dependent PKC and p38 MAPK signaling in the IR heart

Recent advances in RNA-seq have enabled the profiling of gene expression patterns in normal, diseased, or protected myocardia, allowing for the prediction and verification of novel signaling pathways and regulatory mechanisms using molecular biology techniques. In a prior study, we used RNA-seq analysis to identify that Slit2 protein reduces infarct size and maintains contractility in post-IR myocardia by inhibiting inflammatory signaling pathways (Li et al., 2020). Furthermore, RNA-seq analysis results revealed that n-3 PUFAs can preserve mitochondrial structure and function, protect cardiac contractile function, and regulate cardiac myofilaments (Li et al., 2022c). In this study, GSEA predicted that endogenous n-3 PUFAs protect IR hearts by influencing protein synthesis, GPCR signaling, ATP-dependent activity, cellular metabolism, apoptosis, and tissue structure. These findings align with functional measurements, histological examinations, and ATP content analyses. In our exploration of the regulatory mechanisms, we found that PKCδ, PKCε, p38α, PI3K, AKT, and mTOR signaling is mediated by n-3 PUFAs through APLNR inhibition in IR hearts.

In the PKC signaling pathway, upon activation, phospholipase C (PLC) cleaves phosphatidylinositol 4,5-bisphosphate (PIP2) into inositol 1,4,5-triphosphate (IP3) and diacylglycerol (DAG) (Gilman, 1987; Wang et al., 2018). The latter, together with or independently of Ca^2+^, acts as a second messenger to further activate PKC isoforms (Steinberg, 2008). Additionally, p38 MAPK is also regulated by G proteins (Gαs/i/q and Gβγ subunits) and a phosphorylation cascade (Yamauchi et al., 1997; Canovas and Nebreda, 2021). In pathological conditions, PKC isoforms can have both beneficial and detrimental effects on the heart. Detrimental effects include cardiomyocyte death, inflammation, cardiac hypertrophy, and fibrosis (Palaniyandi et al., 2009). In contrast, p38 MAPK is often referred to as a stress-activated protein kinase. It is activated by global ischemia and maintains its activity during reperfusion in isolated rat hearts (Bogoyevitch et al., 1996). p38 MAPK activation is associated with apoptosis in post-IR hearts, while its inhibition reduces the infarct area in post-IR hearts (Bogoyevitch et al., 1996; Ma et al., 1999; Armstrong, 2004). In this study, we observed that PKCδ and p-p38α expression were upregulated in post-IR myocardia/post-HR cells, and this upregulation was suppressed by treatment with elevated levels of endogenous n-3 PUFA/EPA. This suppression was associated with cardioprotective phenotypes in post-IR myocardia. These findings are consistent with those of our previous study, in which the suppressed expression of PKCδ or p-p38α was found in cardioprotection attributed to IR conditioning, CapZ deficiency, and Slit2 overexpression (Yang and Pyle, 2012; Li et al., 2020).

Furthermore, the activation of APLNR by the agonist apelin-13 upregulated PKCδ and p-p38α and downregulated PKCε in *fat-1* IR hearts, and this was associated with suppressed cardiac contractile function, suggesting that the cardioprotection by n-3 PUFAs is negated by APLNR activation under IR conditions. APLNR inhibition by ML221 maintained relatively lower expression of PKCδ and p-p38α and higher expression of PKCε in the WT hearts subjected to IR compared with post-IR hearts without antagonist treatment. APLNR inhibition by EPA in cells also resulted in decreased expression of PKCδ and p-p38α, indicating that APLNR inhibition regulates PKCδ and p-p38α. Furthermore, APLNR activation is linked to cAMP/PKA, PKC, and angiotensin AT1 receptor signaling (Chun et al., 2008).

These results suggest that n-3 PUFAs regulate PKC and p38 MAPK signaling to protect hearts through a G protein-coupled mechanism, namely, APLNR inhibition.

### n-3 PUFA protects the heart by modulating the APLNR inhibition-dependent PI3K-AKT-mTOR signaling axis in the IR heart

Our study, for the first time, demonstrates that n-3 PUFAs protect the heart from IR injury by regulating the PI3K-AKT-mTOR signaling pathway through APLNR inhibition. Activation of the apelin/APLNR system has been shown to regulate the PI3K-AKT-mTOR signaling pathway (Dagamajalu et al., 2022). The PI3K-AKT-mTOR signaling pathway can be activated by GPCR and receptor tyrosine kinase (RTK). When cell surface receptors bind to ligands or undergo conformational changes induced by other factors, PI3K is phosphorylated, catalyzing the conversion of PIP2 into phosphatidylinositol (3,4,5)-triphosphate (PIP3) (Auger et al., 1989). This, in turn, recruits AKT and leads to the phosphorylation of AKT. Activation of the PI3K/AKT signaling pathway results in the phosphorylation of multiple proteins, including mTOR (Hay and Sonenberg, 2004). mTOR is a central component of mTOR complex 1 (mTORC1) and −2 (mTORC2), and in the heart, its major downstream targets include protein synthesis, metabolism, and proliferation, similar to those in other tissues (Sciarretta et al., 2014; Sciarretta et al., 2018). Dysregulation of mTOR is implicated in heart diseases. For instance, mTOR inhibition with Torin 1 increases apoptotic cell death and infarct size in ischemic mouse hearts (Völkers et al., 2013). Cardiac-specific mTOR deletion in mice leads to abnormal activation of AKT, which is associated with cardiac dysfunction and sarcomere disarray (Zhu et al., 2013). Conversely, mTOR overexpression reduces interstitial fibrosis and preserves global cardiac function in post-IR mouse hearts (Aoyagi et al., 2012).

In this study, mTOR signaling was not activated in IR mouse hearts, whereas the PI3K-AKT-mTOR axis was fully activated in IR hearts protected by n-3 PUFAs. IR hearts with activation of the PI3K-AKT-mTOR axis exhibited beneficial phenotypes, including reduced infarction, lower apoptosis rates, and higher myocardial ATP contents, compared with those in IR hearts without mTOR activation. Although we did not verify the downstream targets of the PI3K-AKT-mTOR axis in this study, we can speculate, based on the GSEA and pathological examination, that the PI3K-AKT-mTOR axis contributes to reducing cardiomyocyte death, minimizing myocardial infarction, and maintaining metabolism and morphology.

Furthermore, this signaling axis was not fully activated in the IR hearts or HR cells with upregulated APLNR (i.e., AKT was activated, but not PI3K and mTOR). In contrast, hearts or cells with APLNR inhibition, either through the use of an antagonist (ML221-treated IR hearts) or elevated n-3 PUFA levels (e.g., in *fat-1* IR hearts or EPA-treated HR cells), exhibited synchronized phosphorylation of PI3K, AKT, and mTOR. In protected post-IR hearts, this synchronized activation was associated with changes in myofilament phosphorylation, whereas in protected HR cells, this synchronized activation was associated with improved morphology. Therefore, EPA plays a pivotal role in APLNR inhibition-induced cardioprotection under ischemic conditions.

### Myofilament phosphorylation responds to ALPNR inhibition under IR conditions

In our previous studies, we have demonstrated that the phosphorylation state of myofilaments is sensitive to various stress stimuli in experimental models of mice, pigs, or monkeys, including conditions such as IR, cardioplegia, pressure overload, and a high-fat, high-sugar diet (Yang and Pyle, 2011; Yang and Pyle, 2012; Li et al., 2020; Tan et al., 2021; Zheng et al., 2021). In this study, we have discovered that cardioprotection conferred by endogenous n-3 PUFAs is associated with increased phosphorylation of cTnI, cTnT, and MLC2 in post-IR hearts. These findings align with our previous results, which demonstrated that cardioprotection induced by endogenous n-3 PUFAs is associated with increased or restored phosphorylation of cTnI and several other myofibril proteins under stressful conditions (Li et al., 2022c). Moreover, APLNR inhibition, achieved through the use of the antagonist ML221, influenced the phosphorylation profiles of myofilaments, including increases in the phosphorylation of cTnT and MLC2. Consequently, we investigated how intracellular phosphorylation signaling, mediated by this GPCR, operates in n-3 PUFA-protected IR hearts.

PKC and p38 MAPK are direct regulators of myofilament phosphorylation. PKC translocates and phosphorylates myofilaments upon activation, leading to changes in contractility in an isoform-dependent manner (Steinberg, 2008). Conversely, myofilament phosphorylation targeted by p38 MAPK reduces myofibril contractility (Vahebi et al., 2007), and there are limited insights into the regulation of mTOR on myofilament phosphorylation. Considering that mTOR activation is associated with alterations in cardiac contractility during various physiological and pathological events, it is plausible that mTOR signaling may also impact myofilament phosphorylation. However, this hypothesis requires further confirmation. Moreover, there is cross-talk among these three pathways in ischemic hearts. For instance, Heidkamp et al. reported that the translocation of PKCε and PKCδ leads to the activation of MAPKs (Erk, JNK, or p38 MAPK) (Heidkamp et al., 2001). Additionally, evidence suggests that PI3K is upstream of PKC in ischemic preconditioned hearts (Tong et al., 2000). Further investigation of the interaction of these three pathways will help to understand the regulatory mechanism of apelin/APLNR under IR conditions.

In conclusion, elevated levels of endogenous n-3 PUFAs suppress the increase in APLNR expression induced by IR injury in mouse hearts. APLNR inhibition via ML221 treatment protects myocardial tissues and contractile function in IR hearts by activating the PI3K-AKT-mTOR signaling pathway. To the best of our knowledge, this study is the first to provide mechanistic insights into the role of APLNR inhibition in myocardial IR injury.

## Limitation

This study revealed the cardioprotective effect of inhibition of APLNR in IR injury. However, whether the inhibition of this receptor will enable IR hearts to resist chronic maladaptive remodeling remains to be determined. When developing therapeutics targeting the apelin/APLNR system, further evaluation of APLNR function under pathological conditions such as ventricular pressure overload and myocardial IR is warranted. Although we verified that some signaling pathways/networks are regulated by n-3 PUFAs via ALPNR, our data are limited. As shown in the transcriptomic analysis, complex signaling networks and biological processes are targeted by n-3 PUFAs. Besides, the conclusion of this study are mainly derived from *ex vivo* models; therefore, further *in vivo* and *in vitro* verification remains warranted regarding the signaling regulation and altered biological processes attributed to n-3 PUFAs via APLNR inhibition.

## Data Availability

The datasets presented in this study can be found in online repositories. The names of the repository/repositories and accession number(s) can be found below: GSE223343 (GEO).
